# A Comparison of Nested PCR Assay with Conventional Techniques for Diagnosis of Intestinal Cryptosporidiosis in AIDS Cases from Northern India

**DOI:** 10.1155/2014/706105

**Published:** 2014-01-12

**Authors:** Beena Uppal, Ompal Singh, Sanjim Chadha, Arun Kumar Jha

**Affiliations:** Department of Microbiology, Maulana Azad Medical College and Lok Nayak Hospital, Bahadur Shah Zafar Marg, New Delhi 110002, India

## Abstract

Cryptosporidiosis is a very important opportunistic infection and is responsible for significant morbidity and mortality in HIV/AIDS patients. Although current laboratory methods are generally considered adequate to detect high concentrations of oocysts, they fail to detect cases of cryptosporidiosis in many immunocompromised patients. The present study was done to determine the diagnostic efficacy of modified Ziehl-Neelsen (ZN), antigen detection ELISA, and a nested PCR assay for detection of *Cryptosporidium* in 58 adult AIDS cases with diarrhea from the ART clinic of Lok Nayak Hospital, New Delhi. *Cryptosporidium* was detected in 17 (29.4%), 39 (67.3%), and 45 (77.5%) cases by modified ZN staining, antigen ELISA, and nested PCR assay, respectively. Taking nested PCR as the gold standard, specificity of both modified ZN staining and *Cryptosporidium* antigen detection ELISA was 100% while the sensitivity of the tests was 37.8% and 86.6%, respectively. PCR was more sensitive than the other two diagnostic modalities but required a more hands-on time per sample and was more expensive than microscopy. PCR, however, was very adaptable to batch analysis, reducing the costs considerably. This assay can therefore have considerable advantages in the treatment of immunosuppressed individuals like AIDS patients, allowing their early diagnosis and decreasing the morbidity and the mortality.

## 1. Introduction

*Cryptosporidium parvum* is an enteric protozoan parasite with worldwide distribution which may inhabit the gastrointestinal tract of a wide variety of animals including humans [1, 2]. Infection with *C. parvum *in immunocompetent persons often results in asymptomatic or mild self-limited disease, but in HIV-infected patients, particularly those with low CD4 counts, infection may result in chronic or life-threatening diarrhea, or extraintestinal disease [3–6]. In developed countries, an estimated 14% of AIDS patients with diarrhea have *C. parvum* infection [3]. In developing countries, the parasite is reported in 24% (range: 8.7–48%) of HIV-seropositive adults and children while in India it has been reported in 2–60% of such patients [7–11].

In persons with AIDS, cryptosporidiosis is commonly a permanent diarrheal illness that leads to chronic malabsorption of fluids, nutrients, vitamins, and electrolytes with resulting wasting [12]. This warrants a prompt diagnosis and an early institution of specific therapy to reduce the morbidity associated with the disease. But over the years, detection of this protozoon has become a challenge. Cryptosporidiosis is most commonly diagnosed by identifying oocysts in the stool specimens of infected persons. The diagnostic difficulties arise from the fact that shedding of *C. parvum* oocysts is intermittent even in patients with massive diarrhea [13]. The number of oocysts present in the stool sample may not be adequate for detection and it is usually seen that oocysts are better recovered from watery stools than the formed samples [14]. Modified Ziehl-Neelsen (ZN) staining and fluorescein-tagged monoclonal antibody immunofluorescence (IF) staining techniques are the most commonly utilized diagnostic modalities for intestinal cryptosporidiosis. However the sensitivity of these tests (modified ZN and IF staining) for detecting *C. parvum *oocysts in human stools has been reported to be 10,000 oocysts per g of watery stool, while in formed stools 50,000 or 500,000 oocysts per gram are required for a positive IF or modified ZN staining test, respectively [15]. Therefore, newer and more sensitive techniques are clearly needed to identify these oocysts in the stool specimens.

Coproantigen detection assays and PCR-based methods (nested PCR being more sensitive) have been reported to have a high diagnostic index in such cases. Antigen assays have an advantage of not requiring a skilled microscopist and their specificity has been reported to be high. However, variable sensitivities and specificities have been reported using different kits. The commercially available coproantigen detection ELISA formats use monoclonal antibodies (mAbs) which recognize different sets of surface epitopes and mAbs used in these ELISA kits may not react or react weakly with antigens of different *Cryptosporidium* species. In addition the cost of the test per sample has been reported to be much more than microscopic examination. Thus, ELISA appears to offer no increase in sensitivity over microscopy [16].

PCR based methods have been shown to be more sensitive than the conventional microscopic and immunological methods for detection of *C. parvum *in human feces [17]. Balatbat et al. have reported that the nested PCR assay can identify as few as 500 oocysts per g of stool, which represents a 100-fold increase in sensitivity compared with that of the IF method [18]. This assay may therefore contribute to the identification of patients who are asymptomatic but harbor infection at a threshold below that detectable by the current diagnostic tests.

With the above background in mind, this study was conducted to compare the nested PCR assay for detection of *C. parvum* with the conventional modified ZN staining and antigen detection in stool specimens by ELISA in order to determine the usefulness and practicality of PCR-based methods for diagnosis of *C. parvum* in stool specimens of AIDS patients.

## 2. Materials and Methods

### 2.1. Study Population

Fifty eight adult (>18 years of age) drug naive HIV seropositive subjects fulfilling the WHO case definition of AIDS with or without diarrhea were enrolled from the ART clinic of Lok Nayak Hospital, New Delhi, India. Only those cases with CD4 T lymphocyte counts <200 cells/*μ*L were recruited.

### 2.2. Study Design

A cross-sectional study was conducted from October 2008 to August 2010 on 58 adult HIV seropositive patients in whom the presence of *C. parvum* in the fecal specimens was detected by applying three different diagnostic modalities: modified ZN staining of stool smears, detection of *C. parvum* antigen in the stool by ELISA, and nested PCR for detecting *C. parvum* DNA in the stool sample. The role of nested PCR as a diagnostic modality for detecting *C. parvum* in the stool samples of AIDS patients was evaluated. After an informed consent from the patients, a detailed history regarding the personal details, sociodemographic characteristics, diarrheal episodes, associated signs and symptoms, history of drug intake, exposure history, and so forth were recorded on a pre designed performa. Stool specimens were requested from all the participants.

### 2.3. Specimen Collection and Transport

Fecal samples were collected in clean, wide-mouthed screw capped disposable plastic container and transported to the microbiology laboratory by the patients themselves on the same day avoiding any unnecessary delay.

### 2.4. Laboratory Processing of Specimens

On receipt of the fecal specimen in the laboratory, the sample was divided into two equal portions. From the first aliquot stool smears were prepared, heat fixed, and stained by the Kinyoun's (modified ZN stain) method. Second aliquot was stored at −20°C to perform ELISA and PCR.

One portion of second aliquot of stool specimen was used to detect *C. parvum* antigen by using a commercial ELISA kit for stool samples (IVD Research Inc. CA, USA) according to manufacturer's instructions. This test is a double antibody sandwich in vitro immunoassay for the qualitative determination of *C. parvum *antigen in the feces.

Another portion of second aliquot was used for molecular test. DNA was extracted by using QIAmp DNA stool mini-kit (QIAGEN, Hilden, Germany) according to manufacturer's instructions. A nested PCR approach was used to amplify a 194 bp DNA fragment of *C. parvum* which is located on chromosome 8 and is specific for *C. parvum*. This segment of *C. parvum *DNA as well as the outer primers and the probe correspond to sequences described by Laxer et al. [19]. The PCR method of Mullis and Faloona was used for amplification and the reaction mixture was prepared by the PCR master mix (Fermentas, Canada) containing Taq DNA polymerase and the deoxyribonucleotide triphosphates. The following DNA primers were used:

1. outer primers
   BB-1 (5′-CCGAGT T TGATCCAAA AGTTACGAA-3′)
   BB-2 (3′-ATGATTATTC CGTATACTCC-5′),
2. inner primers
   BB-3 (5′-GCGA AGATGACCTT TTGATTG-3′)
   BB-4 (3′-CCTTGGA CTCTTCTTCT TTAGGGA-5′).

For the first amplification reaction the outer primers, BB-1 and BB-2, were used while for the second round of amplifications the inner primers, BB-3 and BB-4, were used The reactions were performed in a DNA thermal cycler (Mycycler, Bio-Rad, USA) [20]. Thermal-time profiles were the same as described by Balatbat et al. and amplification was done for 35 cycles [18]. Amplification products were visualized by electrophoresing the reaction mixture in 2% ethidium bromide stained agarose gel along with a molecular weight marker. Size markers included in all gels were the 1000 bp DNA ladder (Bio-basic Inc.) The electrophoresis was carried out at a constant voltage of 90 V for 2 hours and a band of 194 bp was taken to be positive result. The bands in the gel were photographed under UV transillumination.

### 2.5. Statistical Analysis

Sensitivity, specificity, positive predictive value, and negative predictive values of different diagnostic techniques were determined by taking nested PCR as the gold standard by using the statistical software SPSS version 17. *P* values were calculated using the Fisher's Exact test.

## 3. Results

Of the 58 study subjects recruited, 44 (75.86%) were male and 14 (24.14%) were females with age ranging between 22 and 61 years (mean age 35.2 ± 4.42 years). The male to female ratio was 3.1 : 1. The predominant age group in males as well as females was 30–40 years followed by 20 to 30 years. Most common mode of HIV acquisition was heterosexual contact (79.4%) followed by injection drug abuse (10.3%).

At the time of enrollment the mean CD4 T lymphocyte count of our study population was 129.2 cells/*μ*L and 45 out of 58 cases (77.5%) had diarrhea. However all subjects had experienced an episode of diarrhea in the last one month prior to enrollment.  Table 1 shows the *C. parvum* detected in study subjects by the various methods and the sensitivity, specificity, and predictive values of the modified ZN staining and antigen ELISA taking nested PCR assay as the gold standard and assuming it to be 100% sensitive and specific.

Table 2 shows the *C. parvum* positivity in study subjects by one or more of the diagnostic modalities. Seventeen samples (29.3%) tested positive for *C. parvum* by all the three methods while 7 (12.1%) samples were positive by nested PCR only. Two samples tested positive for *C. parvum* only by the antigen ELISA and not by the modified ZN staining and nested PCR assay.

**Figure 1:**
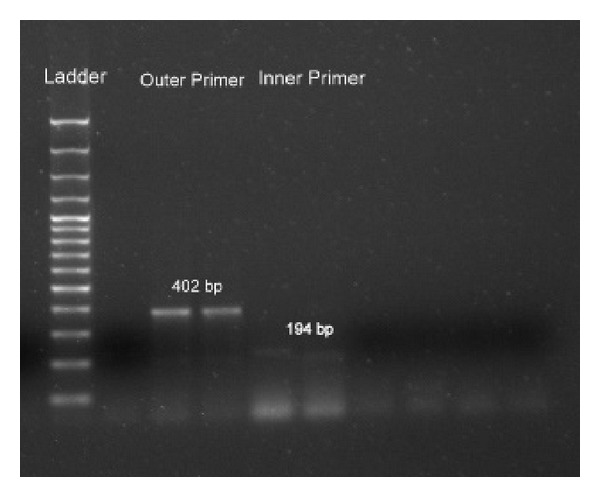
PCR product obtained with the first set of outer primers (402 bp) and the amplicon (194 bp) obtained after amplification with the second set of inner primers.

Figure 1 shows a 402 bp PCR product obtained with the first set of outer primers and the 194 bp amplicon obtained after amplification with the second set of inner primers from DNA extracted from oocysts present in positive fecal specimens.

**Figure 2:**
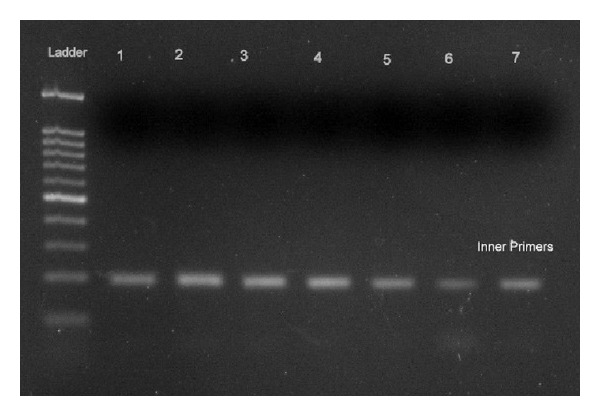
Nested PCR results with the inner primers yielded 194 bp DNA fragment in the positive cases of *Cryptosporidium*. Lane 1: Positive control. Lanes 2–7: Amplicons of positive samples.

Figure 2 shows the nested PCR results with the inner primers that yielded the expected 194 bp DNA fragment in the stool specimens of positive cases of *Cryptosporidium*. Lane 1 is positive control and Lanes 2–7 are the amplicons of positive samples.

## 4. Discussion

Although, with the widespread use of effective antiretroviral therapy (ART), cryptosporidiosis is no longer the devastating illness it once was in AIDS patients in developed countries, it still continues to pose a major threat to AIDS patients in resource-poor, developing countries like India where ART is not widely available or affordable. In patients with AIDS, intestinal cryptosporidiosis may sometimes be lethal and its diagnosis is therefore critically important. But some major pitfalls have been identified in the routine diagnosis of intestinal cryptosporidiosis [21]. Firstly, the direct microscopic examination after modified ZN staining relies on the morphologic recognition of small-sized oocysts which may be scant in number, intermittently shed, or inconsistently stained [13, 22]. This method is therefore impractical to standardize as it is influenced by the individual skills of the microscopist involved. Furthermore the identification of an acid-fast blue-green alga (Cyanobacterium) which is only slightly larger than *Cryptosporidium* and has been associated with a prolonged self-limited diarrheal illness may limit the utility of acid-fast staining in the diagnosis of *Cryptosporidium *[23]. Secondly, the utility of the fluorescence based diagnostic tests is limited by expense and the frequent lack of a fluorescence microscope and trained staff in most of the diagnostic laboratories in developing countries like India. This technique also requires a more hands-on time per sample as batch testing is not possible [24]. *C. parvum *antigen testing by ELISA offers an alternative to the traditional microscopy based diagnostic tests as it is less time consuming and easier to perform and also enables the testing of a large number of samples at one time as batch testing can be undertaken. But the drawbacks associated with this test are the higher cost of the immunoassay kits and the specialist equipment (plate washers and readers) required to automate the whole process. Also the sensitivity and specificity of these tests have been reported to be lower than the immunofluorescence microscopy [25].

PCR technology offers a good alternative to conventional diagnosis of *Cryptosporidium* from both clinical as well as environmental samples [26]. The detection limits reported for PCR based methods by different authors have ranged from 100 to 2,000 oocysts per gram of human feces [27–29]. In this study we describe a sensitive and a specific nested PCR technique for detection of *C. parvum* directly in the stool specimens of AIDS patients.

The sociodemographic data of our study subjects was similar to that reported by some previous Indian studies done on HIV/AIDS cases with diarrhea, in particular the male preponderance, the most common age group of study subjects affected (sexually active young people) and the mode of HIV transmission (heterosexual route being the most common) [7, 30].

The study subjects in the present study (all of which were AIDS cases) had a mean CD4 level of 129.2 cells/*μ*L, while Sehgal et al. reported the counts as 69.66 ± 68.25 cells/*μ*L and Ray et al. as 170 ± 115 cells/*μ*L in AIDS cases in India [31, 32]. This discrepant observation can be attributed to the difference in the stage at which the subjects with AIDS were recruited for the study by the different investigators. 77.5% of our cases had diarrhea which is similar to what has been reported by National AIDS Control Organization in Delhi [33].

In our study specificity of both the techniques that is, modified ZN staining and *C. parvum *antigen detection ELISA was 100%. Modified ZN staining had a sensitivity of 37.8% which is in accordance with previous studies where ZN staining has been found to be 98.9–100% specific with sensitivities ranging from 37–90% [10, 26, 34].* C. parvum *antigen detection ELISA in our study had a sensitivity of 86.6%. Other investigators have reported sensitivities of 66.3–100% and specificities of 93–100% using different kits for the antigen ELISA [16].

In the present study the agreement between microscopy and nested PCR showed that microscopy could identify 37.78% of the cases positive for *C. parvum* whereas ELISA diagnosed 82.22% *C. parvum* positive cases as compared to the nested PCR assay. Nested PCR assay was able to pick up 17.78% more positive cases as compared to modified ZN staining and antigen ELISA. These findings may be explained by the fact that direct microscopy relies on oocysts detection which might not be detectable in clinical samples from all cryptosporidiosis cases, and the absence of oocysts in repeated submissions of samples from symptomatic hosts does not necessarily indicate the absence of infection. In such cases, and particularly when clinical suspicion is high, antigen and/or PCR-based detection methods can be used, as sufficient *C. parvum *antigen or DNA from asexual life cycle forms is present in feces [35]. One of our study subjects had a positive direct microscopy and nested PCR while antigen ELISA was negative whereas two of our cases had a positive result for antigen ELISA and a negative result for modified ZN stain and nested PCR assay. Nonhomogeneous distribution of parasites in stool samples, lack of oocysts in the tested sample, and antigenic diversity among *Cryptosporidium* species explains the poor agreement among these three diagnostic modalities.

## 5. Conclusions

Nested PCR has the potential for accurate diagnosis in HIV seropositive subjects with diarrhea because of its high sensitivity. This will have considerable advantages in the treatment of AIDS patients, allowing early diagnosis before the onset of symptoms. Nested PCR test also has the added ability to directly differentiate between different *Cryptosporidium* genotypes, which assist in determining the source of cryptosporidial outbreaks. Sensitivity, specificity, ability to genotype, ease of use, and adaptability to batch testing make PCR a useful tool for future diagnosis and studies on the molecular epidemiology of *Cryptosporidium* infections. In spite of these advantages its wide spread use is still hindered by its high cost and it remains till now confined to research purposes and epidemiological studies. However there exists a valid explanation for this assay to be routinely used for *C. parvum* diagnosis.

## Figures and Tables

**Table 1:** *C. parvum* detected in study subjects by the various methods and the sensitivity, specificity, and predictive values of the modified ZN staining and antigen ELISA (*N* = 58).

Diagnostic technique	Subjects positive Number (%)	Sensitivity	Specificity	Negative predictive value	Positive predictive value
Modified ZN staining	17 (29.4)	37.8%	100%	100%	31.7%
*C. parvum* antigen ELISA	39 (67.3)	86.6%	100%	100%	68.4%
Nested PCR	45 (77.5)	—	—	—	—

**Table 2:** *C. parvum* positivity in study subjects by one or more of the diagnostic modalities (*N* = 58).

Modified ZN staining	*C. parvum* antigen ELISA	Nested PCR	Total number (%)
+	+	+	17 (29.3)
−	+	+	20 (34.5)
−	−	+	7 (12.1)
+	−	−	0
+	−	+	1 (1.7)
+	+	−	0
−	+	−	2 (3.4)
−	−	−	11 (19.0)
